# The Case of Atypical Sexual Attractiveness in a Male Domestic Dog—A Case Study

**DOI:** 10.3390/ani11113156

**Published:** 2021-11-04

**Authors:** Martyna Woszczyło, Antoni Szumny, Jacek Łyczko, Tadeusz Jezierski, Paulina Krzemińska, Izabela Szczerbal, Marek Świtoński, Wojciech Niżański, Michał Dzięcioł

**Affiliations:** 1 Department of Reproduction and Clinic of Farm Animals, Wroclaw University of Environmental and Life Sciences, Plac Grunwaldzki 49, 50-366 Wrocław, Poland; martyna.woszczylo@upwr.edu.pl (M.W.); wojciech.nizanski@upwr.edu.pl (W.N.); 2 Department of Chemistry, Wroclaw University of Environmental and Life Sciences, C.K. Norwida 25, 50-375 Wrocław, Poland; antoni.szumny@upwr.edu.pl (A.S.); jacek.lyczko@upwr.edu.pl (J.Ł.); 3 Department of Animal Behaviour and Welfare, Institute of Genetics and Animal Biotechnology of Polish Academy of Sciences, Jastrzębiec, 05-552 Magdalenka, Poland; t.jezierski@igbzpan.pl; 4 Department of Genetics and Animal Breeding, Faculty of Veterinary Medicine and Animal Science, Poznan University of Life Sciences, Wolynska 33, 60-637 Poznan, Poland; paulina.krzeminska@mail.up.poznan.pl (P.K.); izabela.szczerbal@mail.up.poznan.pl (I.S.); switonski@jay.au.poznan.pl (M.Ś.)

**Keywords:** semiochemical communication, behavior, sex pheromones, dog, hormones, attractiveness

## Abstract

**Simple Summary:**

The period of heat is a time of naturally increased interest of the male in the female. Males recognizing specific chemical signals are able to find the female in estrus and try to mate with her. According to current knowledge, there is a specific hormonal play accompanying this process, with the rise of estrogens and progesterone in the female thought to be responsible for stimulating sexual arousal in males. In this paper we describe the cases of atypically increased attractiveness in a male, that clearly influenced the behavior of adult, intact males, which made mating attempts during contact with him, even though he had no detectable signs of clinical estrogenization. The “case” animal had a basal level of the hormones considered to be involved in the stimulation of sexual arousal. The case animal was a castrated male with confirmed genetic sex and confirmed lack of gonads, and a urinary chemical profile similar to that of a female in estrus. Even though similar cases are noted in breeding and veterinary practice, to our knowledge this is the first report of cases that include clinical and laboratory examination. As a hypothesis, we propose the involvement of other hormones in the creation of incidental attractiveness, or an increased production of compounds responsible for attractiveness (sex pheromones) resulting from metabolic events unrelated to reproductive processes. Further studies are needed to determine the cause of this phenomenon, which would expand our knowledge about the mechanisms involved in the creation of semiochemical communication and the production of the compounds responsible for the modification of behavior in the signal recipients.

**Abstract:**

During the ovarian cycle in domestic dogs, females do not accept males during the first days of estrus but become attractive to males from the beginning of proestrus, with this attractiveness persisting until the end of the estrus phase. It is believed that increased estradiol is responsible for the female attractiveness to the males. In this paper we describe the case of strong, but atypical attractiveness of a castrated male to various, adult, intact males, influenced by the emitted semiochemical signals. Any significant changes in the level of hormones typically involved in the process connected with estrus and responsible for sexual arousal in the males were assessed. The case animal was a 4 year old castrated male Border Collie that was extremely attractive to various males, which presented high levels of sexual arousal, with intensive sniffing and licking of the preputial area, specific vocalization, increased salivation and, finally, mating attempts. Clinical examination of the castrated male revealed a lack of testes in the scrotum and abdominal cavity confirmed by USG. Laboratory tests indicated basal levels of estradiol, testosterone, and progesterone (15.23 pg/mL, <0.05 ng/mL, 0.25 ng/mL), and sex was confirmed via cytogenetic and molecular analysis. Chemical analysis (HS-SPME) of the urine indicated a huge similarity to the profile obtained from a bitch in estrus, with an elevated level of acetophenone, which has been previously postulated in the literature as being a characteristic of the estrus phase in female domestic dogs. This case presented very atypical sexual attractiveness, particularly when taking into account the basal levels of hormones which, according to current knowledge, are responsible for the creation of attractiveness. As a hypothesis requiring verification, we propose the idea of involvement of other hormones in the creation of incidental attractiveness or increased production of compounds responsible for attractiveness (sex pheromones) resulting from metabolic events unrelated to reproductive processes. To our knowledge it is the first described case presenting this phenomenon, which, with more detailed study, could shed new light on the process of creation of sexual attraction in the domestic dog.

## 1. Introduction

In animals, semiochemical communication plays a crucial role in the creation of sexual behaviors, and apart from the visual stimuli, specific chemical signals are responsible for the induction of sexual interest in the male dogs (Beach 1979). Arousal is usually expressed by behavior including following, approaching, intensive sniffing, licking, salivation, and sometimes vocalization [1,2]. Sniffing is usually focused on the anovaginal region, but the muzzle, ears, and interdigital spaces are also areas of interest [3]. Intensive licking and increased salivation observed during contacting female in estrus, are thought to be connected with an attempt to collect the less volatile molecules that are probably also involved in the process of recognition of current reproductive status [4]. In the female domestic dog, the period of increased attractiveness (proestrus and estrus) takes around 2–4 weeks, with an average of about 21 days [5,6]. The level of attractiveness of bitches may vary depending on the stage of reproductive cycle and some dogs, although they can recognize the first signs of proestrus, do not try to mate females until the so-called optimal time for mating [1]. An abrupt cessation of attractiveness is usually observed during the first days of diestrus and specific vaginal cytological findings are also correlated with this time period [5].

In this paper, we describe the case of unusually increased attractiveness of a castrated male to intact, adult males. This case sheds new light on the development of sexual attractiveness in dogs and indicates the need to conduct in-depth research on the influence of various hormones, and possibly other factors, in the process of estrus development and the formation of sexual attractiveness in dogs.

## 2. Materials and Methods

### 2.1. Case Animal

An owner consulted our Clinic of Reproduction with a case of hyperattractiveness of her 4 year old male Border Collie that had been castrated 3 years earlier. The owner reported that during walks their dog strongly attracted the attention of other males, which presented clearly visible signs of sexual arousal expressed by behavior usually observed during contact with a female in estrus and this condition is persistently present, however, more or less expressed. To evaluate the case, we conducted an attractiveness test, as well as a thorough clinical examination and a broad range of laboratory tests including hormone level evaluation, genetic examination, and the profile of volatile compounds in the urine, assessed using the headspace–gas chromatography/mass spectrometry (HS-GC/MS) technique.

#### 2.1.1. The Animals Used for Attractiveness Test

A group of four, adult, intact Beagle males were used to evaluate the attractiveness of the castrated male. This group of males belonged to the local Experimental Kennel located in the Department of Reproduction and Clinic of Farm Animals at Wroclaw University of Environmental and Life Sciences. Moreover, for the chemical analysis, the urine samples were collected from the case dog as well as two intact and one castrated adult male. All animals were clinically healthy, and dogs used for the attractiveness test were familiar with the procedure of estrus detection including direct contact with females.

#### 2.1.2. Clinical and Blood Examination

During clinical examination of the castrated male, the correct structure of the reproductive organs was assessed. The scrotum was palpated to confirm the lack of testes and ultrasound examination (Esaote MyLab™ 30 Gold, Genoa, Italy) of the abdominal cavity was performed to rule out cryptorchidism. Moreover, the anal sac area was examined and the amount and consistency were checked. For the determination of blood morphology, blood samples were collected by venipuncture of the cephalic vein, into a tube containing an ethylenediamine tetra acetic acid (EDTA). Hematological analyses were performed with the use automated hematology analyzer, Sysmex XN-1000 (Sysmex, Lincolnshire, IL, USA).

#### 2.1.3. Attractiveness Test

To confirm the owner’s reports, an attractiveness test was performed using the four intact males (Beagle dogs 2–4 years old). The castrated Border Collie male was introduced in sequence to each of the testing dogs, one after the other, for contact lasting about two minutes, in a restricted area of the clinic that had not been used previously for semen collection, estrus detection, or any other procedures that the dogs could associate with sexual activity [1,7,8]. To avoid fast, direct confrontation between unknown adult male dogs, the Beagle males were initially kept on the leash to allow contact from a distance. Next, closer contact was allowed so that the dogs could interact directly and sniffing, touching, and licking of each other was possible.

#### 2.1.4. Hormone Level Evaluation

For the determination of estrogen, progesterone, and testosterone levels, blood samples were collected by venipuncture of the cephalic vein, into anticoagulant-free tubes for serum (Separmed^®^). The hormone concentrations in the peripheral blood of the male were determined by an enzyme-linked fluorescence assay (ELFA; mini VIDAS^®^ Biomerieux, Marcy-l’Étoile, France) [9,10]. Hormones were evaluated referencing the basal levels reported by the lab as <20 pg/mL, <1 ng/mL, and <2.5–5 ng/mL, respectively, of estrogens, progesterone, and testosterone.

#### 2.1.5. Cytological Evaluation of the Prepuce Smears

In a female dog, due to the risk of apparent fluctuations in hormone levels resulting from the unreliability of measuring those compounds in very low concentrations, i.e., in the pg range (e.g., estrogens), a confirmatory clinical evaluation of estrogenization of the vaginal wall may also be indicated [10]. To evaluate the potential effect of estrogens, which in estrous females is expressed by the hyperkeratinization of the cranial vagina wall, a cytological evaluation of the preputial mucosa of the male dog was performed [5]. The prepuce smears obtained from the Border Collie were collected by gently swabbing the preputial wall with a cotton-tipped swab [5,11,12]. Diff-Quik staining, usually used for vaginal smears, was used. The cells were classified according to the morphologic criteria used in vaginal smears to visualize any evidence of estrogenization [5,11].

#### 2.1.6. Genetic Tests

Blood samples were collected by venipuncture of the cephalic vein. Cytogenetic analysis was performed on chromosome preparations obtained from a short-term lymphocyte culture. The chromosome complement was studied using Giemsa staining, and the sex chromosomes were identified by their bi-armed morphology. Microscopic evaluation was carried out under a Nikon E600 Eclipse microscope (Melville, NY, USA), equipped with a cooled CCD digital camera and Lucia software. Molecular analysis was performed on DNA extracted from the peripheral blood sample with the use of the commercial Blood Mini kit (A & A Biotechnology, Gdansk, Poland) [13].

#### 2.1.7. Chemical Evaluation of the Urine

Samples of urine were collected from males (the case dog and two intact and one castrated adult male), as well as from a female in estrus as a positive control. The chemical compositions of the samples were determined with the use of the headspace solid-phase microextraction-arrow technique (HS-SPME), and the results were compared between individuals.

Around 400 µL of urine was placed in 10 mL headspace vial together with 2-undecanone (2 µg as emulsion in distilled water) together with 1 mL of brine. Sample was pre-heated at 40 °C for 5 min.

Analyses were performed using an AOC-6000 multifunctional autosampler (Shimadzu, Kyoto, Japan). Around 0.1 g of the sample was placed in a 20 mL headspace glass vial and pre-heated at 40 °C for 5 min. Meanwhile, SPME Arrow (DVB/Carbon WR/PDMS, 2 cm length; CTC Analytics AG, Zwingen, Switzerland) was conditioned at 250 °C for 10 min. Then, the Arrow was exposed above the sample for 30 min.

Exposure was performed with constant shaking (120 rpm) at 40 °C and followed by analyte desorption at 250 °C for 3 min. Analyses were run in triplicates.

## 3. Results

Clinical examination revealed proper construction of the penis and prepuce and lack of other abnormalities which could easily influence the scent of the examined animals like dermatitis or anal gland disorders. The lack of testes in the scrotum or abdominal cavity was confirmed during the ultrasonographic examination. The body conformation (size, weight, shape of the skull), also provided subjective confirmation of the male character of the dog being examined. Blood morphology evaluation revealed that all parameters were within the recommended standards (Table S1).

During behavioral testing, all intact males showed intense sexual arousal during contact with the “case dog”. The signs of increased interest were visible from the beginning, with the testing males strongly pulling on the leash towards the male being examined. This behavior was accompanied by specific vocalization that confirmed strong excitement. When direct contact was allowed on an individual basis, each of the testing dogs presented behavior similar to that usually observed during contact with a female in estrus: intense sniffing of the various parts of the body, but in this case focused mainly on the penile area, with licking of the prepuce area accompanied by strong salivation and high tone vocalization [1,6,14]. No special interest of the area of anal sacs was observed. Finally, attempted mating was observed in all cases. These behaviors were not observed during any contact between the intact male dogs, apart from approaching and short sniffing in some cases.

The results of hormonal evaluation indicated basal levels of the hormones assessed: estrogens 15.23 pg/mL, testosterone < 0.05 ng/mL, and progesterone 0.25 ng/mL. Additionally, cytological evaluation of the prepuce swabs did not reveal any signs of estrogenization, confirming the lack of estrogenic influence. Moreover, no signs of inflammation or infection of this area were detected in the cytological preparations.

The presence of Y-linked and X-linked genes was determined by PCR amplification (SRY), followed by RFLP (ZFX and ZFY), as has been described earlier [13]. The genetic evaluation proved that the dog under examination was a male, and no sex-related genetic disorders were identified.

The profile of the VOCs (volatile organic compounds) isolated from collected urine revealed significant differences between the case dog and the other males, with the case dog’s VOC profile being more similar to that of a female in estrus [15]. Based on retention indices, 18 volatile compounds present in dog urine were identified. Acetophenone, described as a typical compound related to estrus in the female domestic dog, was clearly elevated in both, the examined female and the case dog (Table 1). It was noted that case 1 dog’s urine contained elevated levels of sulfur derivatives, i.e., dithiapentans (20 and ~5% contribution, respectively) as well as previously mentioned acetophenone (19%) compared to reference dogs. Additionally, an elevated amount of propyl mercaptan (~6%) in dog samples (case dog and female in estrus), relative to other control dogs was noted.

## 4. Discussion

Semiochemical signaling is a crucial form of communication between mammals in the context of reproductive behavior [16,17]. Chemical messages specific for the proestrus and estrus, which tend to strongly modify male behavior, are sent by the female in estrus. In domestic dogs (*Canis familiaris*), females become attractive to adult males during proestrus, when the typical, easily observed, clinical symptoms like vulval edema and vaginal bleeding are accompanied by the modification of an emitted VOC [5,6,18]. During proestrus in bitches, the level of estrogens (17 β estrogen) rises from basal 8–15 to 50–100 pg/mL. Whereas, observed at the end of proestrus a decrease of estrogens level is accompanied by an increase of progesterone level, the basal value of which is described as being below 1 ng/mL and during estrus the increase reaches a level from 2.5 ng/mL (around ovulation) to about 30 ng/mL at the end of estrus, and persists elevated for about two months [5]. During estrus, the behavior of the male (as a signal receiver) is modified when in contact with the female, and is characterized by attempts at close approach, sniffing and licking of the anovaginal area in particular, as well as attempted mating [1,6,14]. It has also been reported that some stud dogs are able to recognize the most fertile period of the ovarian cycle and only attempt mating during that time, which could suggest that the semiochemical signals responsible for female attractiveness change during this cycle [1,6]. It is believed that a specific hormonal play involving changes in ovarian hormone concentrations, such as estrogens and progesterone, is strictly responsible for this [5,19,20]. Many papers demonstrate the influence of the reduction of these hormones, as a consequence of ovariectomy, on the specific behaviors of the signal recipients, as well as on the composition of the scent of the females [21,22,23]. Correspondingly, supplementation of estradiol, alone or in combination with progesterone, in spayed females modifies the urine chemical profile towards that of an intact female, as has also been demonstrated in a variety of species [22,24,25,26,27,28,29,30,31].

Apart from physiological events like estrus, some pathological conditions could also be responsible for increased female attractiveness and cause increased arousal in the male. In spayed females, ovarian remnant syndrome (ORS) characterized by the presence of residual ovarian tissue, usually as a consequence of an improperly performed ovariectomy, could be a cause of unexpected interest to males, who exhibit behaviors typical of stud dogs contacting a female in estrus [32,33,34,35]. In those cases, symptoms of estrogenization can be observed months or even years after surgery and are connected to a rise in estrogen secretion by the remnant ovarian tissue. These cases can be easily recognized because the clinical symptoms of estrus are accompanied by the typical (although unexpected after ovariectomy) increase in hormone (estrogens and later progesterone) levels.

Moreover, in females, a prolonged period of attractiveness (e.g., more than 40 days), together with a high frequency of estrus periods, could be caused by the presence of ovarian cysts producing estrogens or hormonally active ovarian tumors like a granulosa cell tumor [36,37].

In males, increased levels of estrogens could be the consequence of feminizing adrenocortical tumors that are clinically manifested by gynecomastia and/or other hypogonadism features [38]. However, Sertoli cell tumors are observed much more often in dogs and are responsible for clinically detectable feminization and for increased attractiveness of the affected male. In those cases, according to Quartuccio et al. [39], approximately 70% of the tumors arise in abdominal testes (cryptorchidism) and, due to their hormonal activity, are responsible for feminizing paraneoplastic syndrome.

It is worth noting that in all of these conditions, an increased estrogen level is the predominant reason for the clinical manifestation of increased sexual attractiveness observed in both males and females. However, in this paper we presented a case in which no clinically detectable signs of estrogenization were observed. The case male with genetically confirmed sex presented a basal level of sex hormones (estrogens, testosterone, and progesterone), lack of gonads, and lack of clinically diagnosed signs of estrogenization (confirmed by a negative result of preputial cytology).

However, when considering the influence of estrogens on attractiveness, it is also worth mentioning that iatrogenic administration of these reproductive hormones also influenced the animal’s behavior. Södersten [40] showed that there was a “marked sex difference in medial pre-optic area estrogen receptor concentrations”, with males having far fewer estrogen receptors in this area than females. This could be an influence on sex differences in behavioral estrogen sensitivity [40]. However, that was not relevant to our case, because no signs of increased acceptance behavior were noted, and more importantly, no increase in estrogen levels was detected.

Even though estrogens, together with progesterone and luteinizing hormone (LH), are the main hormones that increase during proestrus and estrus in dogs and are connected with the behavioral events during estrus, other hormones that are not usually evaluated in clinical practice can also participate in the creation of the complicated hormonal cascade influencing the reproductive status of dogs. A hormone that could be taken into account during future evaluation of similar cases is alpha-melanocyte-stimulating hormone (alpha-MSH), which is thought to be responsible for increased female attractiveness [41].

Alpha-MSH is produced from proopiomelanocortin (POMC), a polypeptide hormone precursor that is expressed in the brain and in peripheral tissues such as the pituitary gland, immune system, and skin [41,42]. In the brain, alpha-MSH is expressed in the hypothalamic arcuate nucleus and in the nucleus tractus solitarius of the brainstem, and it has a crucial role in the regulation of some metabolic functions, like controlling energy balance, thermogenesis, appetite suppression, and, important in the context of our study, contributes to sexual arousal [41]. Although administration of alpha-MSH has been shown to increase the attractiveness of females, in one study, it was shown to increase the attractiveness of odors collected from female rats that had also been treated with estradiol benzoate [43]. The authors also declared that “other pituitary hormones, such as ACTH and prolactin, had no effect on the attractiveness of preputial gland odors of OB-treated hypophysectomized rats”. The exact chemical mechanism for increased attractiveness related to increased alpha-MSH secretion or its artificial administration, is not clearly described, however, based on the methodology of the Agostino and Diano study [41], the increase in attractiveness of the odor secreted by the glands connected with reproductive organs is part of this process. That kind of mechanism could theoretically be involved in the spontaneous increase of attractiveness if, due to not-yet-identified reasons, there was increased production of alpha-MSH. However, taking into account the involvement of alpha-MSH in various metabolic processes including thermogenesis, we cannot exclude the possibility that the mechanism for increased attractiveness is a consequence of modified thermoregulation processes and increased body temperature facilitating evaporation, which could be a factor in increasing the female’s interest, instead of a mechanism including metabolic changes leading to increased production of some chemical attractant. However, it must be clearly stated that these are just theoretical considerations, which need detailed evaluation and further sophisticated studies.

The example of alpha-MSH shows that hormones not routinely examined in reproductive status evaluation could also be taken into account in the search for the agent(s) responsible for the increased attractiveness.

It is worth mentioning that inflammatory conditions like vulvovaginitis in females could be responsible for the increased interest of the male in some cases. What also seems to be important is that, according to the literature, the bacterial flora could be involved in the creation of the final semiochemical signal, thus its alteration could influence the composition of the VOCs emitted by the individual [44,45,46]. From the other side the inflammation by itself could be the reason for modification of the VOCs, for example, present in the urine, what was experimentally confirmed in the study of Gordon [47], in which the induced inflammatory process was the reason for appearance in the urine of the compounds usually not detectable there in humans. Interestingly this compound was acetophenone. However, again, in our study the blood morphology did not confirm any mobilization of the immune system which could suggest ongoing inflammatory processes and suspect the inflammation as a direct reason for increased attractiveness. Additionally, the evaluation of the preputial smears did not reveal the presence of inflammation in this area.

The chemical analysis of the urine collected from the animals revealed that the VOCs profile of the case male was found to be similar to that of a female in estrus, with a high concentration of acetophenone and dithiapentane that was not observed in the control samples collected from other males. The latter compound has been indicated as a putative attractant in brown antechinus [48]. At the same time, acetophenone was identified as one of the factors in semiochemistry in red fox [49]. However, McLean et al. [49] suggested that acetophenone can be the compound used by the foxes for the chemical communication, in this species it probably could not be treated as a sex pheromone, because its presence has been confirmed in individuals of both sexes and without context of estrus. In wolves, the study of Raymer et al. (1984) [50] indicates that acetophenone is connected with the sex and seasonally can be found in the females, but also in castrated males. In our study, however, the castrated male of the domestic dog did not present elevated levels of acetophenone in its urine. Observed increased level of acetophenone could be important also in the context of results presented by Dzięcioł et al. [15], who highlighted acetophenone as one of the characteristic compounds in the urine of estrus canine females correlated with communication of individuals within the species. The results obtained may indicate that the combination of acetophenone and dithiapentane could be a factor influencing attractiveness of the case male and can be responsible for modification of the sexual behavior of the intact adult male dog.

Assuming that, for example, a high concentration of acetophenone (previously confirmed as specific for bitches in estrus) could indeed be responsible for the creation of increased arousal in the males, the question arises as to the mechanism that is responsible for the increased secretion of this substance with the urine.

Even though there is a large number of substances in nature, one substance can be involved in many processes, while another substance can have similar functions in different organisms. Even in the context of semiochemical communication, there are cases where the same compounds have been identified as a sexual attractant in two completely different species. For example, the Asian elephant sex pheromone (Z)-7-dodecen-1-yl acetate is shared with a number of moth species [51].

It is well known that among substances involved in semiochemical communication, differences in concentration can be responsible for the activation of a behavior in the signal recipient. A good example is the aphrodisin protein, which belongs to the lipocalin superfamily and is present in the vaginal secretions of the Golden hamster. It is involved in the process of stimulation of copulatory behavior in males, and its concentration in the vaginal discharge varies at different stages of the ovarian cycle [19].

If acetophenone was the substance responsible for eliciting mounting behavior in dogs, that could explain the increased sexual arousal in the other males when they came into contact with the Border Collie. In fact, special attention during those contacts was observed in sniffing and attempts at licking the prepuce of that dog. In our previous study, the presence of non-volatile compounds that probably had a protein character was postulated to be one of the important elements used in the evaluation of attractiveness by males [4] and proteomic evaluation of the secretions could be indicated in future studies of similar cases.

Another issue that could be taken into consideration is the potential influence of diet on the substances detected in the urine, as a medium full of metabolic products. It could be especially important to analyze this issue, in the context of elevated concentration in the urine of the case male, the sulfate containing compounds. It has been observed that male meadow voles were more attracted to odors from females fed on a high, compared to a low, protein diet [52]. Additionally present in some plants, phytoestrogens can be responsible for the creation of pseudoestrual symptoms, stimulating increased sexual interest in males [53]. However, in the presented case, neither high protein diet nor the presence of phytoestrogens can be considered as the reason for increased attractiveness. The case male was fed with a commercial canine diet, changed from time to time, and no correlation between incidence of attractiveness and particular diet was detected. Additionally used sometimes in dogs, high protein food was not described previously as a factor related to increased attractiveness.

Another issue that must be considered, even though it could not be verified and proven, is the possible effect of hormonal disturbances in the fetal period and the deficiency, in the case of males, of testosterone imprinting of the fetus. Similar to other species, including humans, canine males need exposure to adequate levels of testosterone and its metabolite, dihydrotestosterone (DHT) to develop correctly [6,54]. Success in reproduction is also dependent on the proper exposure to estrogens at time of birth, both for males and females [6,55]. However, that could not be the case in the dogs presented here, because no other male from his litter has shown similar tendencies and features. Moreover, the exact mechanism of the hormonal disorders would be difficult to explain. Even though Savic et al. [56] in their study dedicated to the influence of sex pheromones on an individual’s sexual preference (homosexuality or heterosexuality) found that the brain of homosexual males did not react in the same way to the putative pheromones of the opposite sex, as to same sex semiochemicals, in our case the point was the emission of a signal attractive to other intact males. The case dog did not exhibit any interest in other males and did not respond with acceptance behavior to the approaching males during the test.

When evaluating the influence of exposure to sex hormones before birth, it is also worth mentioning the results of Beach et al. [57], who noticed that females that had received moderate amounts of androgen before birth were more attractive to males, although the level of androgenization (increased doses) did not influence the level of attractiveness.

Mating attempts in mature dogs are connected with increased levels of testosterone and are usually performed in the context of sexual behavior. Sometimes abnormal mounting orientation can be observed in mating attempts with an individual of the opposite sex, which can be a consequence of being raised in isolation and lack of sexual experience. Another aspect of reproductive behavior relates to the social regulation of reproductive activities in domestic dogs, as documented by Cafazzo et al. [58]. Moreover, this kind of behavior can be used to establish a hierarchy and social dominance in the dog’s pack and can be classified as a dominant behavior not directly connected with reproductive activity. In her study dedicated to the issue of reproductive and sexual behavioral problems in dogs, Martens [14] stated that “mounting dogs of the same or the opposite sex can be due to a variety of motivations other than sexual behavior, including play and exploratory behavior.” However, a specific behavior of the males used for the behavioral tests was observed only during contact with the case dog. Otherwise, their behavior was typical for male to male contacts and sexual arousal was not observed in any of these cases.

Kustritz [6] and Martens [14] both mentioned the possibility of the chemical compounds in shampoos containing methyl paraben as a preservative, acting as canine sex pheromones. This could not be the explanation for the case described here, firstly because no products containing these compounds had been used on the case dog, and because other studies have excluded the pheromonal effect of methylparaben in dogs [18,59,60]. Finally, despite the hypothesis of Goodwinn et al. [8], the presence of this compound has been excluded from the group of compounds present in the urine of the bitch in estrus [15,61].

## 5. Conclusions

The described case presents an example of unexplained increased sexual attractiveness of a male dog. The observed symptoms and results of clinical examination clearly show a lack of connection between behavioral events (increased sexual attractiveness) and hormonal play in terms of the hormones typically involved in mammalian reproduction, like estrogens, testosterone, and progesterone. This is a very surprising outcome, given the current knowledge about the mechanisms involved in canine reproduction. Some unpublished reports of similar cases are known to the authors, but the incidental character of this kind of event makes the detailed examination difficult and various features of the individual animals (neutered and intact dogs) creates a problem with finding a common snap point. It could be considered as a possible conclusion, that the increased attractiveness of the case dog was not connected with the typical pathways of reproductive events but was rather a consequence of the accidental production of compounds that increase during estrus, or a consequence of some unidentified metabolic processes. Additional involvement of other hormones like, e.g., alpha-MSH, also described as an element influencing attractiveness, could also be considered. Documentation and detailed evaluation of other similar cases during future studies are suggested to find explanations for this phenomenon.

## Figures and Tables

**Table 1:** Case 1. Profile of volatile organic compounds (VOCs) from various dog urine samples (in percentage of all identified compounds). RT—retention time; CAS—chemical abstract service; KI exp—Kovats retention index.

No	RT	VOCs	CAS	KI Exp.	Case 1 Dog	Female in Estrus	Castrated Male	Intact Male 1	Intact Male 2
1	3.39	Ethyl alcohol	64-17-5	951	8.07	7.93	2.84	3.43	4.61
2	3.5	Propyl mercaptan	107-03-9	859	6.00	5.90	0.34	1.94	1.26
3	4.3	Propane, 1-(methylthio)-	3877-15-4	953	12.64	12.43	17.24	0.71	11.55
4	4.69	2-Butanone	78-93-3	987	0.78	0.77	8.87	8.43	0.79
5	5.56	Butane, 1-(methylthio)-	628-29-5	1078	5.62	5.52	10.65	0.86	13.96
6	5.82	2-Pentanone	107-87-9	1094	2.66	2.62	11.71	19.27	1.32
7	9.06	4-Heptanone	123-19-3	1148	5.10	5.02	0.33	6.72	9.75
8	10.45	2-Heptanone	110-43-0	1189	2.32	2.28	15.32	3.04	1.01
9	11.71	2,4-Dithiapentane	1618-26-4	1387	21.66	21.30	8.39	1.57	14.65
10	13.18	2-Octanone	111-13-7	1291	1.88	1.85	2.17	5.13	0.11
11	14.5	Disulfide, 1-methylethyl propyl	33672-51-4	1391	0.95	0.94	8.61	2.74	3.08
12	18.01	Methyl propyl trisulfide	17619-36-2	1441	0.25	0.24	2.00	7.33	0.16
13	18.49	2,4-Dithiaheptane	4396-19-4	1494	4.84	4.76	0.06	0.79	0.03
14	21.25	2-Decanone	693-54-9	1514	2.53	2.49	3.07	12.15	0.57
15	23.4	Acetophenone	98-86-2	1684	19.50	19.18	0.57	5.16	0.71
16	24.5	Methyl salicylate	119-36-8		1.03	0.73	0.39	16.26	0.83
17	27.9	Phenol	108-95-2	2002	3.08	1.01	5.21	3.06	34.45
18	28.04	Quinoline, 4-methyl	91-63-4	2084	1.10	0.94	2.23	1.42	0.58

## Data Availability

The data that support the findings of this study are available from the corresponding author [M.D.], upon reasonable request.

## Supplementary Materials

The following are available online at https://www.mdpi.com/article/10.3390/ani11113156/s1, Table S1: Blood morphology results of the case animal (SYSMEX XN-1000, Lincolnshire, IL, USA).
