# Supplementary material for: The Case of Atypical Sexual Attractiveness in a Male Domestic Dog—A Case Study
Source: Animals (Basel). 2021 Nov 4;11(11):3156. doi: 10.3390/ani11113156 (PMC8614462; doi:10.3390/ani11113156)
Supplement: Supplementary file 1 [file animals-11-03156-s001.zip › animals-1417636-supplementary.pdf]

Table S1. Blood morphology results of the case animal (SYSMEX XN-1000, Lincolnshire, Illinois, USA**)**

PARAMETER	RESULT	UNITS	RANGE
**WBC**	9,32	G/l	6,00-12,0
**NEU**	6,93	G/l	3,00-9,00
**NEU%**	74,4	%	55,0-75,0
**LYM**	1,26	G/l	1,00-3,60
**LYM%**	13,5	%	13,0-30,0
**MONO**	0,840	G/l	0,150-0,850
**MONO%**	9,00	%	1,00-10,0
**EOS**	0,250	G/l	0,040-0,600
**EOS%**	2,70	%	0,010-6,00
**BASO**	0,040	G/l	0,001-0,100
**BASO%**	0,400	%	0,001-1,00
**RBC**	5,62	T/l	5,50-8,50
**HGB**	172,0	g/l	150,0-190,0
**HTC**	0,512	l/l	0,440-0,550
**MCV**	72,3	fl	60,0-77,0
**MCH**	24,3	pg	21,0-27,0
**MCHC**	336,0	g/l	320,0-360,0
**PLT**	172,0	G/l	150,0-500,0
